# A systematic review of outcome reporting in incisional hernia surgery

**DOI:** 10.1093/bjsopen/zrab006

**Published:** 2021-04-11

**Authors:** D Harji, C Thomas, S A Antoniou, H Chandraratan, B Griffiths, B T Henniford, L Horgan, F Köckerling, M López-Cano, L Massey, M Miserez, A Montgomery, F Muysoms, B K Poulose, W Reinpold, N Smart, NoSTRA HarMoNY

**Affiliations:** Northern Surgical Trainees Research Association (NoSTRA), Northern Deanery, Newcastle Upon Tyne, UK; Northern Surgical Trainees Research Association (NoSTRA), Northern Deanery, Newcastle Upon Tyne, UK; Department of Surgery, Royal Devon & Exeter NHS Foundation Trust, Exeter, UK; Notre Dame University, General Surgery, Murdoch, Western Australia, Australia; Newcastle Surgical Education, Newcastle Upon Tyne, UK; Division of Gastrointestinal and Minimally Invasive Surgery Carolinas Medical Center, Charlotte, North Carolina, USA; Upper Gastrointestinal Surgical Department, Northumbria Healthcare NHSFT, North Shields, UK; Department of Surgery and Centre for Minimally Invasive Surgery, Academic Teaching Hospital of Charité Medical School, Vivantes Hospital, Berlin, Germany; Abdominal Wall Surgery Unit, Hospital Universitario Vall d’Hebron, Universidad Autónoma de Barcelona, Barcelona, Spain; Department of Surgery, Royal Devon & Exeter NHS Foundation Trust, Exeter, UK; Department of Abdominal Surgery, University Hospital Gasthuisberg, KU Leuven, Leuven, Belgium; Department of Clinical Sciences Malmö, Lund University, Malmö, Sweden; Department of Surgery, Maria Middelares, Ghent, Belgium; Division of General and Gastrointestinal Surgery, The Ohio State University Wexner Medical Center, Columbus, Ohio, USA; Department of Surgery and Reference Hernia Centre, Gross Sand Hospital Hamburg, Hamburg, Germany; Department of Surgery, Royal Devon & Exeter NHS Foundation Trust, Exeter, UK

## Abstract

**Background:**

The incidence of incisional hernia is up to 20 per cent after abdominal surgery. The management of patients with incisional hernia can be complex with an array of techniques and meshes available. Ensuring consistency in reporting outcomes across studies on incisional hernia is important and will enable appropriate interpretation, comparison and data synthesis across a range of clinical and operative treatment strategies.

**Methods:**

Literature searches were performed in MEDLINE and EMBASE (from 1 January 2010 to 31 December 2019) and the Cochrane Central Register of Controlled Trials. All studies documenting clinical and patient-reported outcomes for incisional hernia were included.

**Results:**

In total, 1340 studies were screened, of which 92 were included, reporting outcomes on 12 292 patients undergoing incisional hernia repair. Eight broad-based outcome domains were identified, including patient and clinical demographics, hernia-related symptoms, hernia morphology, recurrent incisional hernia, operative variables, postoperative variables, follow-up and patient-reported outcomes. Clinical outcomes such as hernia recurrence rates were reported in 80 studies (87 per cent). A total of nine different definitions for detecting hernia recurrence were identified. Patient-reported outcomes were reported in 31 studies (34 per cent), with 18 different assessment measures used.

**Conclusions:**

This review demonstrates the significant heterogeneity in outcome reporting in incisional hernia studies, with significant variation in outcome assessment and definitions. This is coupled with significant under-reporting of patient-reported outcomes.

## Teaser

This systematic review identifies the current clinical and patient-reported outcomes reported in the literature for incisional hernia.

Significant heterogeneity in outcome reporting, assessment and definitions.

## Introduction

Incisional hernia is a common complication with a documented incidence of 2–20 per cent after open abdominal surgery^1^. The presence of an incisional hernia is associated with significant morbidity and has a considerable impact on patients’ overall quality of life^1^. The management of incisional hernia is complex due to a combination of factors including patient co-morbidity, hernia morphology and the vast array of available surgical options. Consequently, generating a robust evidence base in this cohort of patients is difficult.

The current evidence base in incisional hernia surgery consists of a large number of retrospective cohort studies with a paucity of well designed RCTs^2^. In addition to this, outcome reporting in hernia surgery research is variable. Two systematic reviews examining outcome reporting in inguinal hernia^3^ and ventral hernia^4^ both reported significant heterogeneity between RCTs in defining and reporting clinical outcomes and patient-reported outcomes (PROs).

The European Hernia Society (EHS) has previously issued a series of recommendations regarding outcome reporting in abdominal hernia repair^5^. Following a systematic review and a consultation exercise with international experts, 20 key reporting recommendations were made; 11 of these related to study design, two to hernia morphology, three to operative strategy and four to clinical follow-up. This guideline represents a step towards improving outcome reporting in abdominal wall hernia. However, it has a number of limitations, including the lack of definition to underpin the reporting outcomes, the lack of diversity amongst the expert members of the consultation exercise with no representation of patient perspectives or views and the exclusion of PROs. Current clinical guidelines recommend that surgical repair for incisional hernia should be undertaken to improve patient symptoms and quality of life. Despite this, the majority of studies fail adequately to capture or report patient-centred outcomes such as quality of life and pain. Parker and colleagues reported that four studies out of 31 employed a PRO as a primary endpoint in RCTs assessing ventral hernia repair^6^. To ensure outcome reporting reflects the goals and aims of incisional hernia repair, it is essential that PROs are appropriately defined, captured and reported.

The reporting of standardized definitions and outcomes important to all stakeholders, including clinicians and patients, has been recognized to be of key importance in improving overall outcome reporting and in delivering high-quality clinical research by the Core Outcome Measures in Effectiveness Trials (COMET) initiative. The COMET initiative has developed standard methodology to help formulate core outcome sets (COS) in a variety of clinical scenarios^7^. The aim of a COS is to develop a universally agreed minimum number of key outcomes which are important to all stakeholders and should be reported for all studies in a particular clinical area. A COS in incisional hernia surgery has the potential to overcome a number of the documented limitations of outcome reporting in hernia surgery. The first step towards achieving a COS in incisional hernia repair is to identify the current reported outcomes. The aim of this systematic review was to identify variation in clinical outcomes and PROs currently reported within the literature for incisional hernia.

## Methods

This systematic review was conducted according to a pre-specified protocol based on guidance from the Centre for Reviews and Dissemination^8^ and the Cochrane Handbook^9^ and is reported in line with the PRISMA (Preferred Reporting Items for Systematic Reviews and Meta-Analyses) statement^10^. The protocol was registered with the international, prospective register of systematic reviews, PROSPERO (CRD42018090084).

### Eligibility criteria

All studies reporting clinical outcomes and/or PROs in adults (over 18 years old) undergoing incisional hernia repair between 2010 and 2019 were included. Studies were excluded if clinical outcomes and PROs were reported for other types of hernias (umbilical, epigastric, port-site). In addition, case reports, systematic reviews and letters were excluded.

### Search strategy

The OPID SP versions of MEDLINE (1950 to present), EMBASE (1980 to present) and the Cochrane Central Register of Controlled Trials were searched using the following search terms ‘incisional hernia’, ‘abdominal wall reconstruction’, ‘patient-reported outcomes’ and ‘quality of life’ separated by the Boolean operator ‘AND’. The search strategy was limited to human studies published in English between 1 January 2010 and 31 December 2019. This time frame was chosen to reflect current clinical guidelines from the EHS and contemporary surgical practice, including minimally invasive surgical techniques and enhanced recovery after surgery. Reference lists of included articles were hand-searched to identify any additional studies. All citations were collated within EndNote X7^®^ (Phildelphia, USA) and duplicates were removed.

### Selection of studies

All relevant titles and abstracts were screened by two reviewers (D.H. and C.T.). Full papers of potentially eligible abstracts were retrieved in full. Any conflicts were resolved through discussion with a senior author (B.G.).

### Data extraction

Data extraction was conducted by two reviewers and was verified by a third reviewer. Data were extracted under the broad categories of participant demographics, clinical details, treatment and surgery-related details, clinical outcomes and PROs reported.

All reported outcomes were identified and recorded verbatim. Outcomes were considered defined if text was provided to explain the outcome or a citation was provided to define the outcome. If no explanation or appropriate citation were provided, outcomes were classified as not defined.

### Study quality

Methodological quality assessment of studies included in this review was undertaken using the Risk of Bias In Non-Randomised Studies of Intervention (ROBINS-I) assessment tool^11^ for non-randomized studies and the Cochrane risk of bias tool for RCTs.

### Data analysis

Individual reporting outcomes were extracted and the frequency that each outcome was reported was calculated. The definitions of reporting outcomes were identified and extracted. The consistency of reporting outcomes and the frequency of inconsistencies in definitions of reported outcomes were compared between studies. Descriptive data were expressed using basic statistics including proportions and averages. All data were entered into Microsoft^®^ Excel (Microsoft, Redmond, Washington, USA) for analysis.

## Results

**Fig. 1:**
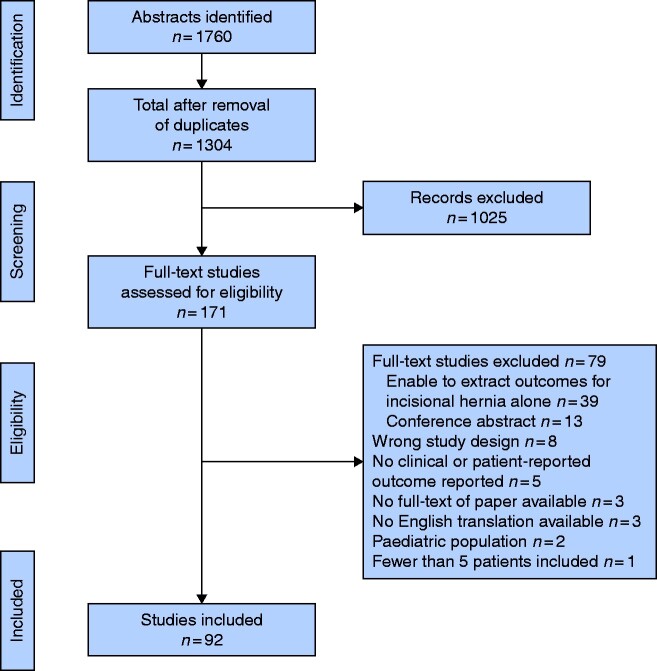
PRISMA diagram showing search strategy

The search strategy identified a total of 1760 manuscripts, of which 456 duplicate references were identified and 1304 studies were screened. Some 171 manuscripts were examined in full, of which 92 studies were eligible for inclusion^12–103^ (*Fig. 1*). Fifteen RCTs (16 per cent), 25 prospective cohort studies (27 per cent), of which 2 (2 per cent) were sub-analyses from RCTs, and 52 retrospective cohort studies (57 per cent) were identified (*Table 1*). Eleven multicentre (14 per cent) and 69 single-centre (86 per cent) studies were included. The primary outcome was defined in 39 studies (42 per cent). Clinical primary endpoints were reported in 30 studies (32.6 per cent), which included complications, hernia recurrence, mesh-related complications (infection or protrusion), structural mesh changes, length of stay, biomechanics and truncal flexion. Patient-reported primary endpoints were assessed in nine studies (10 per cent) which included the SF36 questionnaire^95^, the bodily pain subscale of the SF-36 questionnaire^53^, the physical health subscale of the SF36 at 21 days after surgery^103^, postoperative pain^29^, postoperative pain scores at 3 weeks^54^^,^^104^, Americas Hernia Society Quality Collaborative (AHSQC) pain questionnaire^14^ and HerQLes^14^. Two studies (2.1 per cent) employed a composite primary endpoint of pain and recurrence.^40^^,^^47^

**Table 1:** Study characteristics

Variable	**Number of studies** **(*n* = 92)**
**Study type**	
RCT	15 (16)
Prospective cohort study	25 (27)
Retrospective cohort study	52 (57)
**Multicentre** **Single centre**	11 (14) 69 (86)
**Year of publication**	
2010	8 (9)
2011	5 (5)
2012	5 (5)
2013	12 (13)
2014	11 (12)
2015	16 (17)
2016	12 (13)
2017	11 (12)
2018	5 (5)
2019	7 (8)
**Country**	
Austria	1 (1)
Brazil China	3 (3) 3 (3)
Croatia	1 (1)
Denmark	1 (1)
Egypt	1 (1)
France	3 (3)
Finland	1 (1)
Germany	5 (5)
India	2 (2)
Italy	10 (11)
Japan	1 (1)
Lithuania	1 (1)
Nigeria	1 (1)
Norway	2 (2)
Pakistan	4 (4)
Poland	3 (3)
Romania	6 (7)
Serbia	1 (1)
Spain	4 (4)
Sweden	5 (5)
The Netherlands	6 (7)
Turkey	8 (9)
UK	6 (7)
USA	12 (13)

Values in parentheses are percentages.

## Outcome reporting

**Fig. 2:**
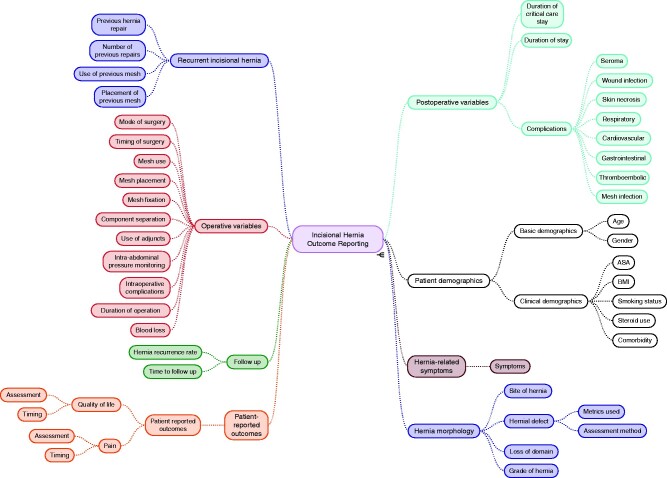
Outcomes extracted

A total of 2215 outcomes were reported, these outcomes were mapped to eight broad-based domains (*Fig. 2*), including patient and clinical demographics, hernia-related symptoms, hernia morphology, recurrent incisional hernia, operative variables, postoperative variables, follow-up and PROs.

### Study bias

**Fig. 3:**
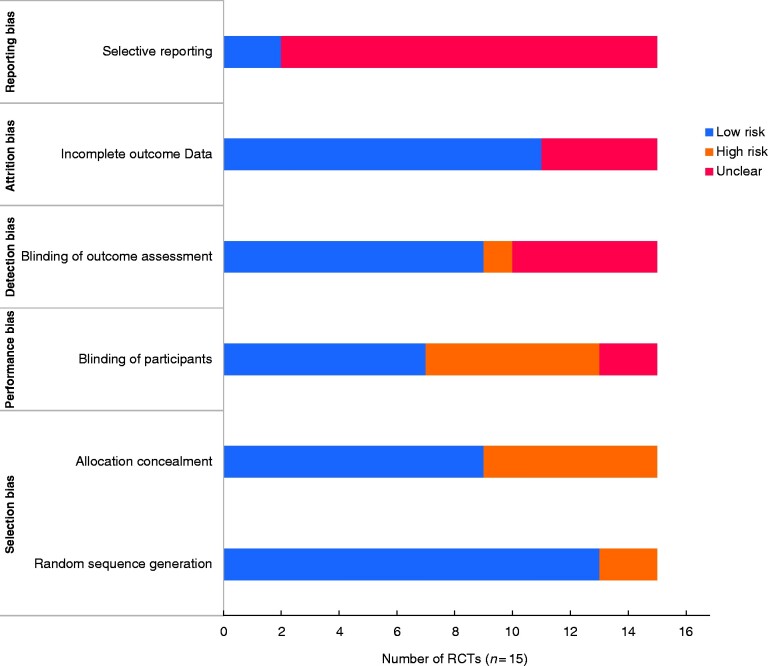
Study bias according to the Cochrane risk of bias tool for RCTs

**Fig. 4:**
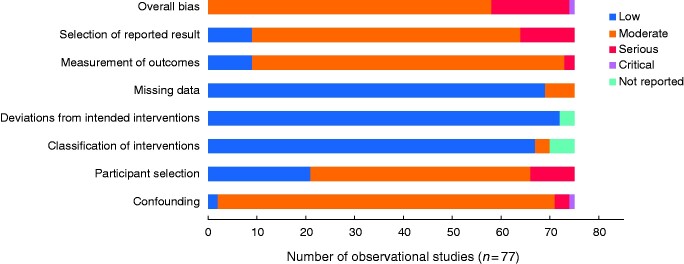
Study bias according to the ROBINS-I risk of bias domain for observational studies

The risk of bias associated with the RCTs is outlined in *Fig. 3* and with observational studies is outlined in *Fig. 4*. The risk of bias was high in six RCTs, with the majority of bias being attributed to the lack of blinding of outcome assessors and selective outcome reporting. For observational studies, 17 studies were identified as being seriously or critically biased, with the domains associated with the highest risk of bias being participant selection, measurement of outcomes and selection of reported outcomes.

## Patient and clinical demographics

Outcomes were reported in 12 292 patients undergoing incisional hernia repair. Patient demographics were well reported, however, clinical demographics relating to BMI, ASA classification and co-morbidity were less well reported (*Table 2*). Patient co-morbidity was reported in 48 studies (52 per cent), of which chronic obstructive pulmonary disease was the commonest co-morbidity reported. Co-morbidities were often reported in an ad hoc manner, with only one study employing a systematic approach by using the APACHE system^65^. Important clinical demographic details regarding steroid use and smoking status were poorly reported, with 17 (18 per cent) and 33 (36 per cent) studies reporting these outcomes respectively. Smoking status was defined in 12 studies (13 per cent), with significant variation in the definition employed (*Table 3*).

**Table 2:** Patient and clinical characteristics

Patient and clinical characteristics	**Frequency reported** **(*n* = 92)**
**Age**	86 (93)
**Gender**	87 (95)
**BMI**	64 (70)
**ASA**	34 (37)
**Smoking status**	33 (36)
**Steroid use**	17 (18)
**Co-morbidity**	48 (52)

Values in parentheses are percentages.

**Table 3:** Definitions of smoking status

Definition of smoking status	Frequency reported
**>5 pack years**	1 (1)
**Active smoker**	3 (3)
**Chronic smoker**	1 (1)
**Nicotine use prior to 6 weeks before hernia repair**	1 (1)
**Patient self-reported**	3 (3)
**Tobacco use**	1 (1)
**Current smoker and ex-smoker**	2 (2)

Values in parentheses are percentages of the total number of studies (*n* = 92).

## Hernia-related symptoms

Symptomatic indications for surgical intervention were reported in 12 studies (13 per cent): six prospective cohort studies, one RCT and five retrospective cohort studies (*Table 4*). A number of composite symptoms were often reported, with studies failing to discriminate between different hernia-related symptoms, treatment strategy and outcomes. Two studies used validated quality-of-life measures to assess hernia-related symptoms^14^^,^^16^. The majority of studies reported hernia-related symptoms in the elective setting, with only one study reporting symptomatic indications in the emergency setting^25^.

**Table 4:** Reporting of hernia related symptoms

Author	Hernia symptoms
**Poelman *et al*.** ^51^	Complaint of bowel obstruction (patients felt pain or had difficulties passing stool) or aesthetically unpleasing
**Rogmark *et al*.** ^53^	Cosmesis, pain, movement restrictions, bowel symptoms, episodes of incarceration
**Lauscher *et al.*** ^105^	Discomfort, occasional sensation of pressure, continuous pain and pain on pressure
**Akinci *et al*.** ^25^	Incarceration was defined as irreducibility of an external hernia and strangulation as irreducibility with objective signs of ischaemia or gangrene at operation procedure
**Schoenmaeckers *et al.*** ^106^	Pain
**Ah-kee *et al*.** ^24^	Pain, discomfort, cosmetic complaints and functional disability
**Saeede *et al*.** ^107^	Pain and swelling
**Aliotta *et al*.** ^14^	Americas Hernia Society Quality Collaborative pain questionnaire and HerQLes questionnaire
**Feng *et al*.** ^16^	HerQLes questionnaire
**Gillion *et al*.** ^15^	Study-specific quality-of-life questionnaire

## Hernia morphology

Significant variation exists in the reporting and assessment of hernia morphology (*Table 5*). The site of the hernia was reported in 25 studies: six prospective cohort studies, three RCTs and 16 retrospective cohort studies. Recommended EHS guidelines for the classification of incisional hernia were only used in seven studies. A total of 15 prospective cohort studies, 10 RCTs and 35 retrospective cohort studies reported hernia defect size, using a variety of definitions. The most common definition used was defect surface area, with 11 studies reporting this metric. Surface area was calculated using the length and width dimensions of the hernia, with these measurements assessed during the operation in two studies, clinically in the supine position in two studies and using preoperative CT imaging in two studies. Twenty-five studies reported the mode of assessment of hernia defect size with preoperative clinical assessment reported by four studies, CT in 10 studies, with one study reserving CT for use in hernia defect size greater than 10 cm, CT or ultrasound scan (USS) in two studies, USS in two studies and intraoperative assessment in 11 studies. Loss of domain was reported by six studies. A total of 12 studies reported on grade of incisional hernia, with three prospective studies and five retrospective studies employing the Ventral Hernia Working group classification.

**Table 5:** Reporting of hernia morphology

Hernia morphology variable	**Frequency reported** **(*n* = 92)**
**Site of previous incision**	20 (22)
**Site of hernia**	25 (27)
**Hernial defect**	60 (65)
**Modality of hernia detection**	
Clinical	4 (4)
CT	10 (11)
CT/USS	2 (2)
USS	2 (2)
Intraoperative	11 (12)
**Loss of domain**	6 (7)
**Grade of hernia**	12 (13)

Values in parentheses are percentages. USS, ultrasound scan.

## Recurrent incisional hernia

Recurrent incisional hernia outcomes were reported by 37 studies: 11 prospective cohort studies, three RCTs and 23 retrospective cohort studies. The number of previous incisional hernia repairs were documented by 12 studies alone, with detail on mesh use provided in 16 studies and mesh placement in 12 studies. Use of previous component separation was reported in five studies. Use of adjunctive measures, such as botox or preoperative pneumoperitoneum, was reported in four studies.

## Operative variables

Operative variables were reported in the majority of studies, with mode and timing of surgery reported in 83 (90 per cent) and 42 (46 per cent) studies respectively (*Table 6*). The majority of studies (21 per cent) reported outcomes on open incisional hernia repair (57 studies, 62 per cent), 19 studies reported outcomes on laparoscopic incisional hernia repair, 15 (16 per cent) reported on both open and laparoscopic repair and one (1 per cent) reported on a hybrid procedure. Elective hernia repair was reported in 25 studies (27 per cent), emergency repair in three (3 per cent) and a combination of elective and emergency in six (7 per cent). Operative technique, including mesh use, mesh placement and fixation, were reported by the majority of studies. Outcomes pertaining to use of specialist techniques, including component separation (24 studies, 26 per cent), use of adjuncts (8 studies, 9 per cent) and intra-abdominal pressure monitoring (2 studies, 2 per cent), were poorly reported.

**Table 6:** Reporting of operative variables

Operative variable	**Frequency reported** **(*n* = 92)**
**Mode of surgery**	83 (90)
**Timing of surgery**	42 (46)
**Mesh use**	88 (96)
**Mesh placement**	84 (91)
**Mesh fixation**	69 (75)
**Use of component separation**	24 (26)
**Use of adjuncts**	8 (9)
**Use of intra-abdominal pressure monitoring**	2 (2)
**Intraoperative complications**	12 (13)
**Duration of operation**	45 (49)
**Blood loss**	6 (7)

Values in parentheses are percentages.

## Postoperative outcomes

Postoperative morbidity was reported by 82 studies (89 per cent) (*Table 7*), with this outcome defined as 30-day morbidity in 17 studies (18 per cent). Generic postoperative complications, including respiratory, cardiovascular, thromboembolic and gastrointestinal, were reported by a third of studies. Specific postoperative complications were more widely reported, with wound infection (68 studies, 74 per cent) and seroma formation (60 studies, 65 per cent) being the most commonly reported. Skin necrosis and mesh infection were significantly under-reported, with only 12 (13 per cent) and eight (9 per cent) studies respectively, reporting these outcomes.

**Table 7:** Reporting of postoperative outcomes

Postoperative variable	**Frequency reported** **(*n* = 92)**
**Duration of critical care stay**	2 (2)
**Duration of stay**	62 (67)
**Postoperative complications**	82 (89)
**Seroma formation**	60 (65)
**Wound infection**	68 (74)
**Skin necrosis**	12 (13)
**Respiratory complications**	23 (25)
**Cardiovascular complications**	10 (11)
**Gastrointestinal complications**	31 (34)
**Thromboembolic complications**	16 (1)
**Mesh infection**	8 (9)
**Grade of postoperative complications**	17 (18)
**Postoperative mortality**	27 (29)
**Re-operation rate**	31 (34)
**Re-intervention rate**	25 (27)
**Readmission rate**	7 (6)

Values in parentheses are percentages.

Significant variation was demonstrated in defining postoperative complications. Wound infection was defined in 10 studies, with a total of eight different definitions employed (*Table 8*). Four different definitions for seroma formation were identified (*Table 9*). Of the five studies reporting mesh infection, only two studies provided a comprehensive definition. Complications were graded in 17 studies, with 12 studies providing detail on classification/grading systems. Postoperative mortality was reported in 23 studies (29 per cent), with two studies documenting the time frame in which this outcome was measured. Re-operation and re-intervention rate were reported in 31 (33 per cent) and 25 (28 per cent) studies respectively.

**Table 8:** Reported definitions for wound infection

Author	Definitions
**Peres *et al*.** ^50^	Infectious cellulitis, treated with local measures and changing antibiotics
**Bittner *et al*.** ^108^ **Kaafarani *et al*.**^98^ **Westphalen *et al*.^101^**	Centers for Disease Control and Prevention classification
**Strâmbu *et al*.^57^**	Deep wound infection defined as purulent suppuration
**Pajtak *et al*.** ^49^	Redness or purulent wound secretion
**Rogmark *et al*.** ^53^	Superficial SSI defined as clinical signs of cutaneous wound infection in need of antibiotic or bedside treatment; deep SSI as wound infection surgically drained in the operating ward
**Ion *et al*.** ^65^	Superficial infections occurred mainly during the early postoperative period (the first 10–14 days); they generally evolved in the subcutaneous dead space associated with a serohematic collection and had a favourable evolution after the application of conservative measures. Deep juxtaprosthetic infections, with a significant local and general response, required wide opening of the prosthetic bed, repeated antiseptic lavage in the focus, targeted antibiotic therapy and exhibited a favourable slow progress over 3 to 8 weeks
**Mommers *et al*.** ^38^	Wound complications were reported as surgical site occurrences, defined as any wound complication (haematoma, superficial and deep wound infection, abscess, seroma, fistula and wound dehiscence). Infectious wound complications were reported separately as SSIs, defined as abscess, infected seroma, superficial or deep wound infection
**Moreno-Egea *et al.*** ^40^	Wound infection was defined as redness, discharge of pus from the wound or a positive bacterial culture

SSI, surgical site infection.

**Table 9:** Reported definitions for seroma formation

Author	Definition
**Rogmark *et al*.** ^53^	A fluid accumulation in need of aspiration/surgical intervention
**Munegato *et al*.** ^42^	Morales–Conde classification
**Pajtak *et al*.** ^49^	Ultrasound scan proven
**Moreno-Egea *et al*.** ^40^	Seroma was defined as a fluid collection detected by palpation on clinical examination when patients attended for routine follow-up clinic appointments

## Follow-up

Time to follow-up was reported in 81 studies (88 per cent), with a variety of time points reported for follow-up ranging from 14 days to 137 months. Hernia recurrence was the only long-term outcome reported in the majority of studies, with recurrence rate reported in 80 studies (87 per cent) (). A total of nine different definitions for detecting hernia recurrence were identified, of which four were based on clinical examination, one on patient or physician reporting, two on combined clinical and radiological examination and two on radiological examination alone (Table 10).

**Table 10:** Reporting for hernia recurrence

Modality of detection for hernia recurrence	**Frequency reported** **(*n* = 92)**
Clinical	7 (8)
Clinical defined as any palpable or detected fascial defect located within 7 cm of the hernia repair	1 (1)
Clinical and CT	12 (13)
Clinical – a recurrent hernia was diagnosed when a fascial defect could be palpated when lifting the head from the examination table to raise the abdominal pressure	1 (1)
Clinical/USS/CT	3 (3)
CT	4 (4)
Defined as a hernia discovered by clinical examination at 1 year	1 (1)
Patient/professional reported	1 (1)
USS	3 (3)

Values in parentheses are percentages. USS, ultrasound scan.

## Patient-reported outcomes

PROs were evaluated in 31 studies (34 per cent): 11 prospective cohort studies, nine RCTs and 11 retrospective cohort studies. A variety of patient-reported outcome measures (PROMs) were used, including ten ad hoc measures, two disease-specific measures and eight generic measures (*Table 11*). Fourteen studies were designed as cross-sectional studies assessing PROs as a one-off assessment ranging from 3–49 months after surgery. Sixteen studies were designed as longitudinal studies. Thirteen studies provided baseline data and only three studies provided long-term data beyond 3 years.

**Table 11:** Patient-reported outcomes in incisional hernia surgery

Patient-reported outcome measure	**Frequency** **(*n* = 92)**
**Ad hoc PROMs**	
Better quality of life after the procedure	1 (1)
Discomfort	1 (1)
Discomfort, cosmesis, functional disability	1 (1)
Fatigue and movement restriction	1 (1)
Foreign body sensation and rigidity of the abdominal wall	1 (1)
Satisfaction	3 (3)
Improvement on visual analogue scale 0–10	1 (1)
Return to full activity	1 (1)
Return to everyday activities (e.g., a walk, driving, making family visits, attending social meetings)	1 (1)
Patient satisfaction – Swedish Hernia Register	1(1)
Not reported	3 (3)
**Disease-specific PROMs**	
Carolina Comfort Scale	3 (3)
HerQLes	2 (2)
**Generic PROMs**	
FACT-G	1 (1)
EuroQoL-5D	1 (1)
SF36	11 (12)
Daily Activities Questionnaire	1 (1)
WHO Quality of Life – BREF	1 (1)
Quebec back pain disability scale	1 (1)
Activities Assessment Scale	1 (1)
Derriford Appearance Scale	2 (2)
**PROMs assessment – study design**	
Cross-sectional	14 (15)
Longitudinal	16 (17)
Not reported	1 (1)
**Pain assessment measurement tools**	
Americas Hernia Society Quality Collaborative pain questionnaire	1 (1)
Pain scale 0–10	1 (1)
Numerical Analogue Scale 0–10	3 (3)
Visual Analogue Scale	10 (11)
Visual Analogue Scale 0–10	6 (7)
Visual Analogue Scale 0–100	1 (1)
Visual Numeric Scale	1 (1)
Most patients report pain or an unpleasant sensation when bending over, due to the stiffness of the mesh	1 (1)
Pain was defined as following: mild (no use of analgesics), moderate (daily use of non-steroidal anti-inflammatory drugs or weak opioids) or severe (daily opioid use).	1 (1)
Do you have any on going pain?	1 (1)
Chronic pain	1 (1)
Not reported	5 (5)
**Pain assessment – study design**	
Cross-sectional	14 (15)
Longitudinal	23 (25)
Not reported	2 (2)

Values in parentheses are percentages. PROM, patient-reported outcome measure.

Pain was evaluated in 39 studies (42 per cent): 17 prospective cohort studies, nine RCTs and 16 retrospective cohort studies. Pain was assessed at variable time points using a variety of measurement tools. The majority of studies assessing pain employed a pain scale, however a total of seven different types of pain scale were used to assess pain scores. Only one study used a disease-specific assessment measure to assess pain-related outcomes^14^. Twenty-three studies were designed as longitudinal studies, with 15 studies providing baseline data and two studies providing long-term data at 5 years.

## Discussion

Incisional hernia surgery is a complex surgical entity, which requires comprehensive assessment and planning prior to selecting the correct operative strategy to improve patient symptoms and quality of life whilst limiting morbidity and reducing the risk of recurrence. Study reporting pertaining to incisional hernia should reflect all component parts of this process of clinical decision making and management. At present reporting standards for incisional hernia do not appropriately reflect this complexity. There is a huge emphasis on reporting clinical outcomes, with the most commonly reported outcomes being mesh use and placement, hernia recurrence and postoperative complications, with significant under-reporting of patient selection criteria, hernia morphology and PROs. Coupled with this there is significant heterogeneity in the manner in which they are defined, with multiple differing definitions for a number of key outcomes.

Hernia recurrence was a primary endpoint in 14 studies and was reported overall in 80 studies (87 per cent). Despite this outcome being widely reported, the majority of studies failed to define how recurrence was defined; of the 32 studies (35 per cent) that defined hernia recurrence, a total of nine definitions were identified. Only one of these nine definitions relied on patient- and/or professional-led reporting of recurrence. The remaining studies used a mixture of differing clinical assessment and radiological evaluation to determine recurrence. Postoperative complications were also widely reported, with 82 studies (87 per cent) reporting this outcome, however, the time frame in which this outcome was assessed was reported by only two studies, and the severity of complications by 17 (18 per cent). There is significant under-reporting of important postoperative complications, such as wound infection, seroma formation and mesh infection. Furthermore, when these outcomes are reported, there is significant heterogeneity in the definitions employed to determine their incidence. Wound infection was reported in 68 studies (74 per cent), of which nine studies defined this outcome measure using a total of eight different definitions. Wound infection has previously been documented to be poorly reported in surgical trials, both as a primary and secondary endpoint, due to a lack of standardized definition^109^. This consequently reduces the measurement properties of this outcome due to the variation in definition across studies, thus leading to variable detection rates of the outcome.

There is significant under-reporting of important preoperative factors regarding optimal patient selection and indication for surgical intervention. More than half of the identified studies included in this review failed to report co-morbid status, BMI, ASA grade, smoking status and steroid use. These important parameters are recognized risk factors for adverse events in incisional hernia repair^110–112^ and help decision making and risk stratification when considering the optimal operative strategy in incisional hernia repair. The lack of detail regarding patient physiology and co-morbid status fails to provide clear guidance on patient selection, thus limiting the clinical application of the obtained results, and further contributing to the heterogeneity observed in clinical practice.

PROs are of particular relevance and importance in the benign disease setting whereby the main goals of treatment are to improve quality of life and symptom control. This is not reflected in the current literature for incisional hernia surgery, with only a third of studies reporting PROs, pain-related outcomes and hernia-related symptoms. Furthermore, a PRO was the primary endpoint in only nine studies. In contrast, a third of studies reported a clinical outcome as the primary endpoint, thus highlighting the greater emphasis on clinician-centric outcomes in this cohort of patients. Coupled with the under-reporting of PROs, there are significant methodological drawbacks associated with the design of the studies reporting these outcomes. These include the lack of use of validated, disease-specific measures for hernia surgery combined with the lack of baseline and longitudinal data and consistent assessment time points. The Carolinas Comfort Scale (CCS) and the HerQLes measures were the only disease-specific measures identified to assess quality of life and were used in only five studies. The CCS is a well designed, validated outcome measure for use in patients undergoing hernia repair with mesh^113^. The HerQLes has been designed and validated for use in patients undergoing ventral hernia repair^114^. The Americas Hernia Society Quality Collaborative pain questionnaire was the only disease-specific measure used to assess pain in patients undergoing ventral hernia repair^115^. Disease-specific measures have the ability to detect subtle differences between patient and treatment groups, which generic measures may potentially miss^116^. The use of hernia-specific PROMs should be advocated when reporting PROs in this cohort of patients. Designing methodologically robust studies with appropriate PRO assessment is key to better outcome reporting. Alongside this, high-quality reporting of PROs is paramount to the clinical interpretation and utility of these complex outcome measures.

A number of clinical guidelines exist within the field of incisional hernia repair^5^^,^^117^^,^^118^. Unfortunately, there is poor penetrance of these guidelines in clinical and academic practice; only seven studies (8 per cent) employed the EHS guidelines when classifying incisional hernias. Eight studies (9 per cent) employed the Ventral Hernia Working Group guidance to grade incisional hernias. These classification systems represent unidimensional aspects of incisional hernia surgery and are often based on expert opinion and current published literature, which often reflect clinical and surgical outcomes alone. The development of a multidimensional system, which reflects the opinions of all key stakeholders, including surgeons, patients, radiologists and methodologists, is more likely to have better clinical and academic utility.

The development of a COS in incisional hernia has a number of advantages, including standardization of outcomes reporting through the inclusion of a minimum number of key outcomes in all incisional hernia-related research which are of equal importance to patients and clinicians. This will in turn lead to transparent, consistent and robust reporting between studies. A COS in incisional hernia can be used in a number of ways, including in clinical and epidemiological studies, in RCTs, integration into current national and international hernia registries and to aid robust evidence synthesis and comparison through systematic review and meta-analysis. Standardizing reporting outcomes methodology will help strengthen the evidence base in incisional hernia surgery and will aid the development and delivery of future high-quality research.

The main limitations of the present study include the inclusion of literature limited to the English language; this may have led to missing important outcomes relevant to an international audience. The second key limitation is that assessment of the current evidence was limited to published literature and did not include grey literature, including published guidelines. This may have a potential impact on the outcomes identified in this systematic review.

## Funding sources

Funding was provided by the British Hernia Society and European Hernia Society.
